# Complete Genome Sequences of Micrococcus luteus Strains NCCP 15687 and NCCP 16831, Isolated in South Korea

**DOI:** 10.1128/MRA.01558-19

**Published:** 2020-02-27

**Authors:** Sanghyun Lee, Ye-Won An, Chi-Hwan Choi, Mi-Ran Yun, Suyeon Kim, Hyangmin Cheong, Young Sill Choi, Dae-Won Kim

**Affiliations:** a National Culture Collection for Pathogens, Pathogen Resource TF, Center for Infectious Diseases, Korea National Institute of Health, Cheogju-si, Chungbuk, South Korea; University of Maryland School of Medicine

## Abstract

In this study, the complete genome sequences of Micrococcus luteus strains NCCP 15687 and NCCP 16831 were determined and deposited in the National Culture Collection for Pathogens (NCCP) of South Korea. Genomic DNA was isolated from blood samples from patients infected with M. luteus.

## ANNOUNCEMENT

Micrococcus luteus is a Gram-positive to Gram-variable bacterium found in a wide range of habitats, such as soil, dust, air, and the human body (1–3). M. luteus is an opportunistic pathogen for nosocomial infections in immunocompromised patients (4). In this study, the complete genome sequences of M. luteus strains NCCP 15687 and NCCP 16831 were determined and deposited in the National Culture Collection for Pathogens (NCCP), which has been run by the Korea National Institute of Health (KNIH) for collecting, managing, and distributing the pathogen resources isolated in South Korea. NCCP 15687 and NCCP 16831 were isolated from blood samples from M. luteus-infected patients in Jeonju, South Korea, in May 2010 and in Jinju, South Korea, in June 2012, respectively.

M. luteus strains were cultured for 16 to 24 h at 37°C in 5% sheep blood agar medium (blood agar plates, product no. 500101; Korea Synergy Innovation) under anaerobic conditions. Genomic DNA was extracted from blood samples by digestion with proteinase K and 10% SDS, followed by purification with phenol and precipitation with ethanol. The bacterial genomes were sequenced using a combination of PacBio RS II and Ion S5 sequencing technologies to improve sequencing accuracy. Briefly, PacBio RS II sequencing was performed as follows. Qualified genomic DNA was fragmented using g-TUBES (Covaris) and end repaired to prepare SMRTbell DNA template libraries (fragment sizes of >10 kb were selected using the BluePippin system) according to the manufacturer’s instructions (Pacific Biosciences, USA). Single-molecule real-time (SMRT) sequencing was performed with an RS II sequencer (Pacific Biosciences), using standard protocols (MagBead Standard Seq v2 loading; 1 × 180-min movie) based on P4-C2 chemistry. Ion S5 sequencing was conducted as follows. A DNA library was constructed according to the protocol of the Ion Xpress Plus fragment library kit. The prepared library was sequenced with an Ion S5 sequencer (Ion Torrent, USA), using Ion 530 OT2 reagent. RS II HGAP v3.0 assembly was performed for high-quality *de novo* assembly using a single PacBio library. High-quality Ion S5 reads were used in the program proovread v2.14.0 (5) to correct potential sequencing errors in the PacBio long reads.

For NCCP 15687, a total of 54,822 and 7,850,134 high-quality reads were obtained by PacBio RS II and Ion S5 sequencing, with 222.6-fold and 493.9-fold coverage, respectively. For NCCP 16831, a total of 95,703 and 9,705,852 reads were obtained by PacBio RS II and Ion S5 sequencing, with 309.5-fold and 484.1-fold coverage, respectively. The genome size of NCCP 15687 (2,445,333 nucleotides; G+C content, 73.1%) was similar to that of NCCP 16831 (2,459,530 nucleotides; G+C content, 73.0%). The genomes of the two strains shared 98.3% similarity, as determined by OrthoANI v3.1.1b (6). Automated annotation of the genome was performed using the NCBI Prokaryotic Genome Annotation Pipeline (PGAP) (7). A total of 2,391 genes, including 2,167 protein-coding genes and 58 RNAs, and a total of 2,271 genes, including 2,117 protein-coding genes and 57 RNAs, were predicted from the genomes of NCCP 15687 and NCCP 16831, respectively. Moreover, Roary v3.5.0 pangenome analysis (8) with default parameters identified 1,813 orthologous genes in the two strains. The genomic characterization and evolutionary pattern of these strains will provide further information about other M. luteus strains and their virulence genes in humans.

### Data availability.

The data obtained have been deposited in GenBank under accession no. CP043849 and SRA accession no. SRR10803019 (PacBio) and SRR10762780 (Ion S5) for NCCP 15687 and under accession no. CP043842 and SRA accession no. SRR10803018 (PacBio) and SRR10762802 (Ion S5) for NCCP 16831.
